# Probing oxygen activation on plasmonic photocatalysts

**DOI:** 10.3389/fchem.2022.988542

**Published:** 2022-09-12

**Authors:** Fons Dingenen, Rituraj Borah, Rajeshreddy Ninakanti, Sammy W. Verbruggen

**Affiliations:** ^1^ Department of Bioscience Engineering, University of Antwerp, Sustainable Energy, Air & Water Technology (DuEL), Antwerp, Belgium; ^2^ Nanolab Center of Excellence, University of Antwerp, Antwerp, Belgium; ^3^ Department of Physics, Electron Microscopy for Material Science, University of Antwerpen, Antwerp, Belgium

**Keywords:** surface plasmon resonance (SPR), photocatalysis, probe, Kinetics, oxygen activation

## Abstract

In this work we present an assay to probe the oxygen activation rate on plasmonic nanoparticles under visible light. Using a superoxide-specific XTT molecular probe, the oxygen activation rate on bimetallic gold-silver “rainbow” nanoparticles with a broadband visible light (> 420 nm) response, is determined at different light intensities by measuring its conversion into the colored XTT-formazan derivate. A kinetic model is applied to enable a quantitative estimation of the rate constant, and is shown to match almost perfectly with the experimental data. Next, the broadband visible light driven oxygen activation capacity of this plasmonic rainbow system, supported on nano-sized SiO_2_, is demonstrated towards the oxidation of aniline to azobenzene in DMSO. To conclude, a brief theoretical discussion is devoted to the possible mechanisms behind such plasmon-driven reactions.

## 1 Introduction

Direct plasmonic photocatalysis is a process in which plasmonic metal nanoparticles act as both the catalytic active site as well as the light absorber simultaneously. Recent reports have shown that it is possible to use visible light to fuel different chemical transformations on plasmonic nanoparticles directly, without the requirement of a photoactive semiconducting substrate (Brongersma et al., 2015; Zhou et al., 2021). These include the degradation of different organic dyes like methylene blue (Chen et al., 2012; Pincella et al., 2014; Boerigter et al., 2016) and sulforhodamine-B (Zhu et al., 2009; Chen et al., 2010), mineralization of formaldehyde (Chen et al., 2008; Kale et al., 2014), selective reduction of various nitroaromatics to form their corresponding azo-compounds (Zhu et al., 2010), ethylene epoxidation (Christopher et al., 2011), selective oxidation of alcohols (Zhang et al., 2012), esterification of aldehydes and alcohols (Zhang et al., 2014), selective CO_2_ hydrogenation (Zhang et al., 2017; Inoue et al., 2019; Yu and Jain, 2019) and cross-coupling reactions (Wang et al., 2013; Xiao et al., 2014b; Zhao et al., 2016). More recently, further tuned hybrid systems occurred, where several metals are alloyed or used in a core-shell configuration, even with less plasmonic, more catalytically active metals (e.g., Ru, Pt, and Pd) in antenna-reactor systems (Swearer et al., 2016; Robatjazi et al., 2020; Zhou et al., 2020). Apart from its renewable character, the use of sunlight allows chemical transformations to take place at ambient temperature and pressure. This substantially reduces equipment cost and increases catalyst operation time due to the less harsh reaction conditions (Lang et al., 2014; Peiris et al., 2016; Wu et al., 2017). The Surface Plasmon Resonance (SPR) effect can also selectively channel photon energy, ultimately facilitating the rate-limiting step (Kale et al., 2014).

Most of the oxidative chemical reactions as those listed above, involve activation of oxygen as a key step in the reaction mechanism (Chen et al., 2010; Wittstock et al., 2010; Christopher et al., 2011; Zhang et al., 2012; Huang et al., 2014). Traditionally, these oxidation reactions are carried out using stoichiometric quantities of toxic inorganic reagents that often entail a high waste disposal cost. Alternatively, by solely using molecular oxygen as primary oxidant, a high atom economy and low waste cost can be achieved. In addition, no hazardous inorganic reagents are needed (Punniyamurthy et al., 2005; Campbell and Stahl, 2012; Ali et al., 2014; Lang et al., 2014). It has already been demonstrated that oxygen can be activated on plasmonic nanoparticles by exploiting the SPR effect, where excited ‘hot’ electrons are formed by visible light illumination that are subsequently transferred to O_2_ molecules adsorbed on the nanoparticle surface, as studied by surface-enhanced Raman spectroscopy (SERS) (Huang et al., 2014; Wang et al., 2014; Da Silva et al., 2015; da Silva et al., 2016), electron paramagnetic resonance spectroscopy (EPR) (Gao et al., 2014; Long et al., 2014; Sakamoto et al., 2015; Di et al., 2016; Wen et al., 2016), UV-VIS spectroscopy (Pasparakis, 2013; Wen et al., 2016), phosphorescence spectroscopy (Vankayala et al., 2011, 2013; Gao et al., 2014), density functional theory (DFT) calculations (Christopher et al., 2011; Zhao et al., 2014, 2016) and kinetic isotope experiments (Christopher et al., 2011).

In this study a wet-chemical probing technique is presented to monitor plasmonic oxygen activation directly in the reaction solution, a convenient experimental tool that has not been applied in this context before. To that end, XTT {2,3-bis(2-methoxy-4-nitro-5-sulphophenyl)-5-[(phenylamino)carbonyl]-2H-tetrazolium, sodium salt} is used as a probe molecule to examine the charge transfer process of hot electrons to dissolved oxygen. XTT is readily reduced by superoxide anions (O_2_^−^) to form its corresponding formazan dye, that shows a strong visible light absorption (Oritani et al., 2004; Li et al., 2013; Azócar et al., 2019). This distinct color change associated with the tetrazolium/formazan redox couple, forms the basis of the existing applications as indicators in cell biology (Seidler, 1991; Berridge et al., 2005; Popescu et al., 2015). One of these applications is the quantitative detection of superoxide anions (O_2_^−^), formed by the one-electron reduction of O_2_, in biological systems. Tetrazolium salts are therefore commonly used in cell ageing or toxicity studies, as reactive oxygen species play an important role in such processes. Another application is as indirect assay of the enzyme activity of superoxide dismutase (Sutherland and Learmonth, 1997; Oritani et al., 2004). It has also been applied to detect superoxide in different non-biological systems such as on irradiated TiO_2_ (Goto et al., 2004; Popescu et al., 2015), fullerenes (Yamakoshi et al., 2003; Hotze et al., 2008) or to study chemiluminescence in carbon nanodots (Xue et al., 2011), amongst others. The present work shows the applicability of the XTT assay in (broadband) plasmonic catalysis to assess the oxygen activation rate during the reaction. By carefully evaluating the impact of operational parameters such as irradiation intensity, and photobleaching rate, a kinetic model is applied that enables a facile, quantitative estimation of the oxygen activation efficiency. As a proof of concept, the direct plasmonic photocatalytic oxidation of aniline to azobenzene (AB) is used to evaluate the oxygen activation efficiency of a broadband plasmonic rainbow photocatalyst.

## 2 Materials and methods

### 2.1 Plasmonic rainbow photocatalyst synthesis

The rainbow photocatalyst synthesis is already described in previous work (Verbruggen et al., 2016; Dingenen et al., 2021). In short, colloidal Au_x_Ag_(1-x)_ nanoparticles of different sizes and compositions (x varying from 0.2 to 1) were prepared following a modified Turkevich synthesis (Turkevich et al., 1951). Here, appropriate amounts of 0.01 M HAuCl_4_·3H_2_O (Sigma–Aldrich, > 99.9%) and 0.01 M AgNO_3_ (Sigma–Aldrich, > 99%) precursor solutions were mixed, stirred vigorously and brought to boil. Then, 1 ml of a freshly prepared 1 wt% sodium citrate (Sigma–Aldrich, 99%) solution was quickly added as stabilizer and reducing agent. After boiling for 30 min, the reaction mixture was quenched in an ice bath. The rainbow sample was prepared by mixing equal amounts of the nine different colloidal solutions. The plasmonic bimetallic nanoparticles were subsequently deposited on silica nanopowder (Sigma-Aldrich, primary particle size 12 nm, 99.8%) by photoimpregnation. An appropriate amount of SiO_2_ nanopowder was added to the colloidal rainbow solution under vigorous stirring and UVA illumination (Philips Cleo UVA, 25 W, 365 nm) for 90 min, so a total nominal metal loading of 1.5 wt% was achieved. The resulting suspension was centrifuged (15 min; 10,000 g), washed and dried overnight at 278 K. The supernatant after centrifugation was completely colorless, indicating all nanoparticles were effectively deposited on the SiO_2_ substrate. UV-VIS absorption spectra of the colloidal nanoparticle solutions were recorded using a Shimadzu UV-VIS 2600 double beam spectrophotometer. The powder samples were characterized on the same apparatus, equipped with a 60 mm BaSO_4_ coated ISR-2600 integrating sphere. For more detailed characterization of the bimetallic rainbow nanoparticles themselves (size determination, electron microscopy and elemental analysis), we kindly refer the reader to our previous study (Verbruggen et al., 2016).

### 2.2 XTT assay

To monitor the oxygen activation efficiency (O_2_^−^ formation) of the SiO_2_ supported pure plasmonic rainbow photocatalyst, XTT was used as a probe molecule as it reacts with superoxide anions to form its corresponding colored formazan dye. 5 mg of the photocatalyst powder was suspended in a XTT sodium salt solution (10 ml, 0.1 mM, Sigma-Aldrich, ≥ 90%) in DMSO (dimethyl sulfoxide, Emplura, 99.0%) in a glass reactor vessel containing a screw cap (total volume of 11.3 ml) with airtight septum. The resulting suspension was purged with oxygen (5 min, 50 ml min^−1^) to ensure oxygen saturation. The reaction vessel was subsequently illuminated with solely visible light using a 300 W Xe source (Oriel Instruments) equipped with a 420 nm cut-on colored glass long-pass filter. At different time intervals, 0.5 ml aliquots of the reaction mixture were taken and centrifuged at 14,800 rpm for five minutes to ensure complete catalyst separation. The resulting supernatant was analyzed using UV-VIS. The intensity of the Xe source was measured with a calibrated intensity spectrometer (Avantes Avaspec 3648). Absolute irradiance was subsequently calculated by integrating the intensity from 400 nm–800 nm.

### 2.3 Plasmon mediated oxidation of aniline

In a typical experiment, catalyst powder (25 mg) was suspended in an aniline solution (10 ml, 0.5 mм, Sigma-Aldrich, ≥ 99.5%) in DMSO in the airtight glass reaction tube. The resulting suspension was purged with O_2_ (5 min, 50 ml min^−1^) to ensure the reaction mixture was saturated with oxygen. The reaction vessel was subsequently illuminated with solely visible light using the same light source and filter as described above. During the reaction, 0.5 ml aliquots were taken at different time intervals and centrifuged for five minutes at 14,800 rpm to completely separate the catalyst. The resulting supernatant was analyzed by UV-VIS spectroscopy.

## 3 Results and discussion

### 3.1 Plasmonic photocatalyst

**FIGURE 1:**
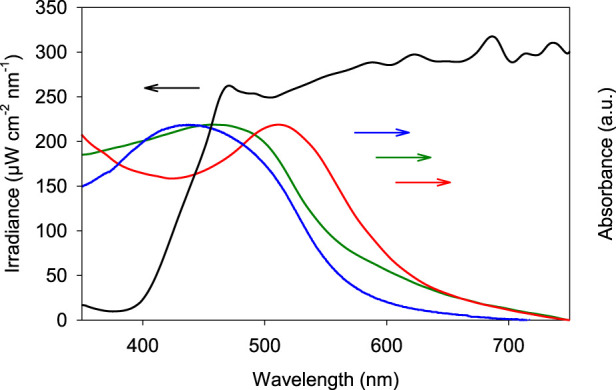
Intensity profile of the 300 W Xe lamp equipped with a 420 nm cut-on filter (black curve), liquid UV-VIS absorption spectrum of the unsupported colloidal rainbow mixture in water (blue), powder UV-VIS absorption spectrum of the plasmonic rainbow sample on SiO_2_ (green) and the plasmonic rainbow sample on SiO_2_ suspendend in DMSO (red).

A plasmonic broadband “rainbow” system is synthesized based on earlier work that demonstrated the simultaneous absorption of multiple wavelengths over a broad range of the visible light spectrum (Verbruggen et al., 2016). In brief, the system consists of Au_x_Ag_(1-x)_ bimetallic nanoparticles with x ranging from 0.2 to 1. Equivalent amounts of these particles are mixed together to obtain the rainbow sample, that is characterized by a broad visible light absorption band (Figure 1). The particles are deposited on a non-photoactive SiO_2_ support (12 nm nanopowder, Sigma-Aldrich) at a total metal loading of 1.5 wt%. Although it has been shown that unsupported plasmonic nanoparticles can catalyze reactions, their stability under these conditions remains troublesome, hence the reason for selecting SiO_2_ as support. Furthermore, it strongly facilitates recovery and recyclability of the catalyst post-reaction (Lang et al., 2014; Xiao et al., 2014a). Figure 1 shows the diffuse reflectance UV-VIS absorption spectra of the resulting supported rainbow catalyst on SiO_2_. After depositing the nanoparticles on SiO_2_ a red-shift is observed that can be explained by the higher refractive index of the SiO_2_ support compared to water. In this work we used SiO_2_ nanopowder with a crystallite size of 12 nm as the support. This way the resulting catalyst can be finely dispersed in liquid media at low concentrations, even enabling direct UV-VIS absorption measurements without the need for particle separation. Figure 1 also shows the absorption spectrum of the rainbow catalyst suspended in dimethyl sulfoxide (DMSO), that is used as solvent in both the probe reaction for determining the oxygen activation efficiency as well as the oxidation reaction of aniline. Because of the difference in refractive index between DMSO, water and air (n_DMSO_ > n_water_ > n_air_) the SiO_2_ supported rainbow photocatalyst suspended in DMSO experiences an additional red-shift, thereby well overlapping with the intensity spectrum of the light source (300 W Xe source with 420 nm cut-on filter, Newport). For more detailed characterization of the bimetallic rainbow nanoparticles themselves (size determination, electron microscopy, elemental analysis, etc.), we kindly refer the reader to our previous publications, as this is not the scope of the present work (Verbruggen et al., 2016).

### 3.2 XTT probe reaction and kinetic model

In this work, XTT is used to probe plasmonic oxygen activation by a broadband plasmonic rainbow photocatalyst. The experiments are conducted in DMSO, since superoxide is considerably more stable in DMSO compared to water. DMSO is an aprotic solvent that readily deactivates water, thus preventing the unwanted reaction between the superoxide anion radical and any trace amounts of water that may be present (Che et al., 1996; Oritani et al., 2004; Hayyan et al., 2012).

**FIGURE 2:**
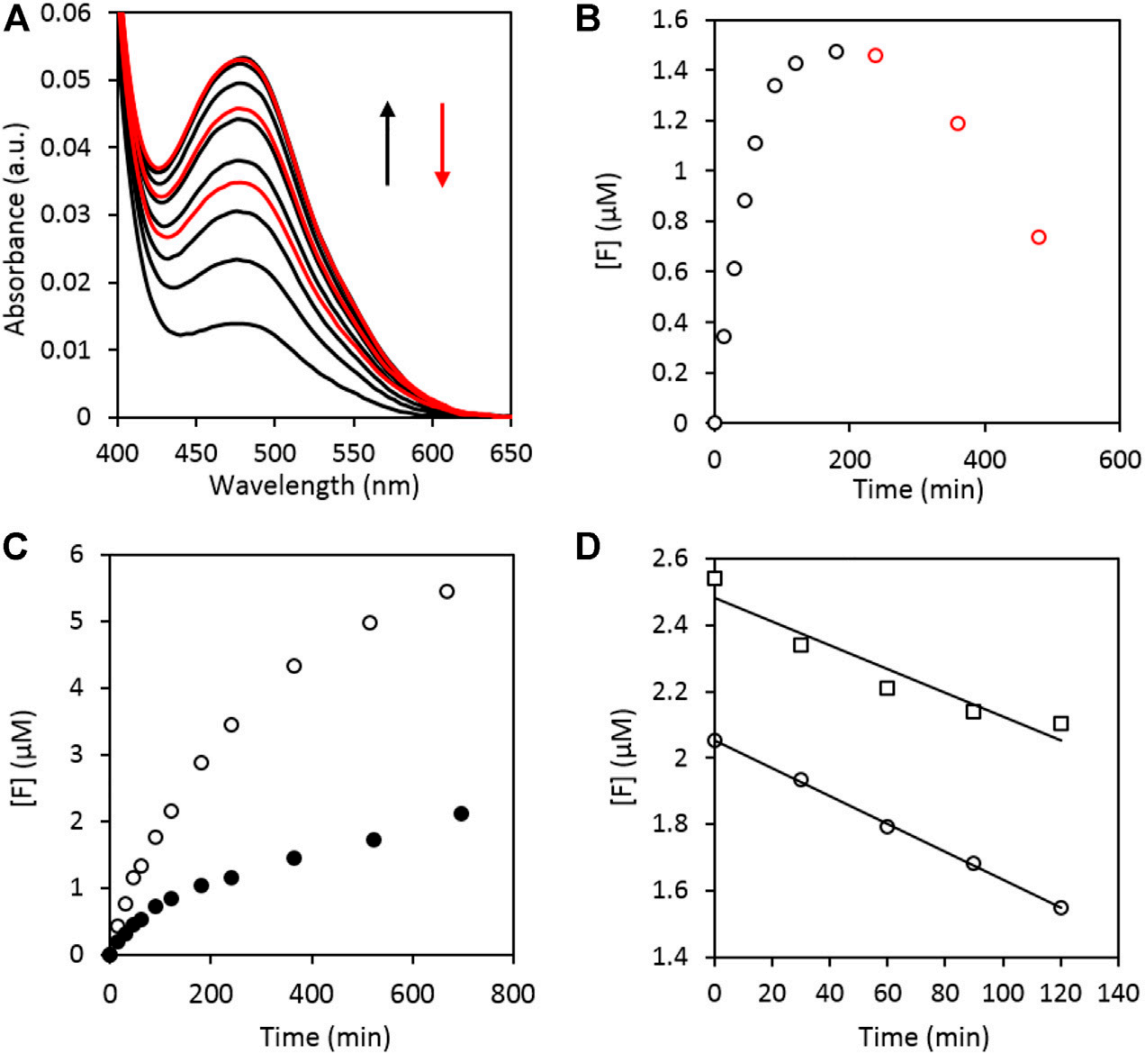
**A)** UV-VIS absorption spectra of the illuminated reaction mixture taken at different time intervals showing the formation of XTT formazan with λ_max_ at 480 nm. The absorbance increases during the first 180 min of illumination (black curves) after which the absorption band starts to decrease again (red curves). **(B)** Time dependent change of the formazan concentration ([F]) of **(A)**. The values were corrected for the initial conversion occurring during sample preparation. **(C)** Difference in formazan production between an illuminated (open data points) and dark (filled data points) sample. **(D)** Zero order discoloration of the formed formazan illuminated at 81 mW cm^−2^ [○, *R* ^2^ = 0.994, slope = (4.18 ± 0.08) nM min^−1^] and 53 mW cm^−2^ [□, *R* ^2^ = 0.912, slope = (3.4 ± 0.8) nM min^−1^].

An exploratory assay using the SiO_2_-supported plasmonic catalyst under VIS illumination is shown in Figure 2. The formation of a colored formazan-product with a maximum absorbance at 480 nm is clearly observed (Figure 2A) (Sutherland and Learmonth, 1997; Oritani et al., 2004). The conversion to the corresponding formazan concentration was achieved by applying Beer’s law. Here, the molecular absorption coefficient of XTT-formazan in DMSO at 480 nm was estimated to be (26.1 ± 0.3).10^3^ M^−1^ cm^−1^, based on literature (Paull et al., 1988), after correcting for the solvent. A detailed description of the estimation is given in the supporting information. The time-dependent change in the formazan concentration ([F]) is depicted in Figure 2B. A blank experiment with pristine SiO_2_ in a XTT/DMSO solution under illumination showed no XTT to formazan conversion. The reduction of XTT to its colored derivate can therefore be attributed to the presence of the metallic nanoparticles. Interestingly, the bottom curve of Figure 2A shows that already some formazan is formed during sample preparation (*i.e.* even before illumination) invoking the hypothesis that the noble metal bimetallic nanoparticles already have a basal oxygen activation activity, without illumination. This is confirmed for Au nanoparticles in dark by Stowers et al*.* and Xu et al*.* (Stowers et al., 2013; Xu et al., 2014) They also observed an additional increase in oxygen activation by alloying gold and silver. To verify this hypothesis, two additional experiments were performed where one reaction vessel was illuminated, while the other was kept in the dark (Figure 2C). It is clear that formazan is in fact readily formed without illumination, albeit to a far lesser extent than when the sample is illuminated. It can thus be concluded that the visible light induced plasmon excitation improves the oxygen activation efficiency (i.e. O_2_^−^ formation) compared to its dark counterpart. These results are in agreement with a study on α-Al_2_O_3_ supported silver nanocubes (60 nm edge length) by Christopher et al. (Christopher et al., 2011). Due to SPR-mediated oxygen activation, they observed a four-fold increase in the ethylene epoxidation rate when the catalyst was illuminated.

An interesting observation is that in Figures 2A,B the formazan concentration first levels off and subsequently decreases (approximately after 180 min of illumination, black versus red). The stability of the formed formazan under illumination was therefore investigated in more detail. A formazan solution was illuminated at different intensities in the absence of any catalyst. The results are given in Figure 2D, where a zero-order discoloration can be observed.

The observed conversion of XTT to formazan and its subsequent photoinduced discoloration can be described more in detail by a kinetic model. The model is derived in detail in the Supporting Information section, and results in Eq. (1):

$$\left[F\right]=\left({\left[XTT\right]}_{0}-k_{3}t\right)\left(1-e^{-k^{\prime }t}\right)$$ (1)

With [F] the formazan concentration, *k*_*3*_ a zero-order discoloration rate constant, and *k’* an apparent first order rate constant, obtained by assuming that that [O_2_^−^] >> [XTT]. [F] can be easily monitored using the absorption band at 480 nm. In addition, assuming no discoloration of the formed formazan would take place, the formazan concentration at t = infinity equals [XTT]_0_ as all XTT would be converted into formazan. [XTT]_0_ can therefore be substituted by the absorbance of formazan at 480 nm at *t* = infinity (in the absence of discoloration), ultimately yielding Eq. (2) that can be monitored exclusively based on UV-VIS data.

$$\left[F\right]=\frac{Abs_{480\;nm}\left(t=t\right)}{\epsilon_{DMSO,\;480\;nm}\;.\;l}=\left(\frac{Abs_{480\;nm}\left(t=\infty \right)}{\epsilon_{DMSO,\;480\;nm\;}.\;l}-k_{3}t\right)\left(1-e^{-k^{\prime }t}\right)$$ (2)

With ε_DMSO, 480 nm_ the estimated molecular absorption coefficient for formazan and l the path length of light through the solution (1 cm).

**FIGURE 3:**
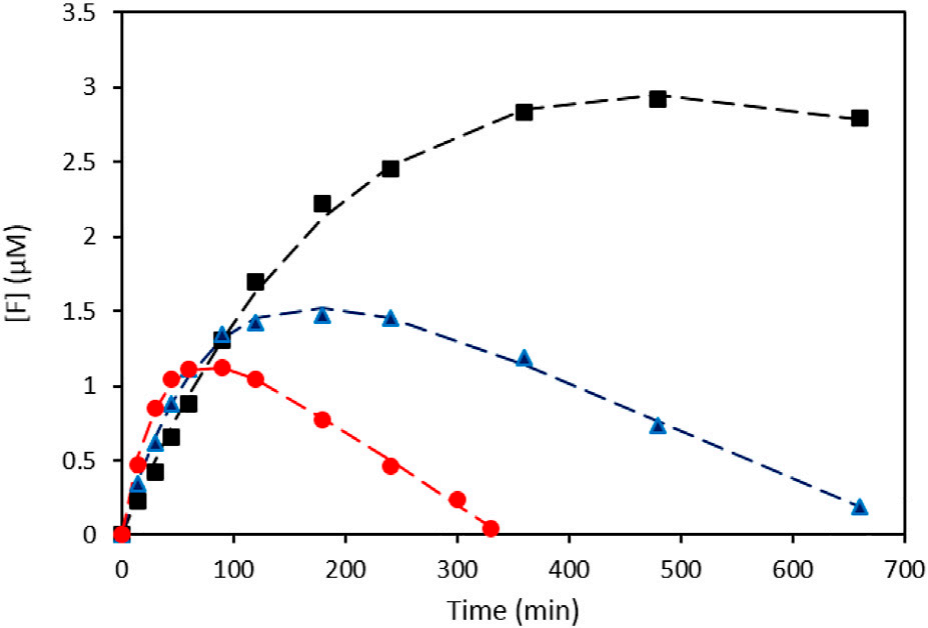
Formazan concentration ([F]) as a function of illumination time for three different intensities and their corresponding model fits. (black ■: 56 mW cm^−2^; blue ▲: 101 mW cm^−2^; red ●: 455 mW cm^−2^).

In Figure 3 the model predictions (dashed lines) are compared to the experimental data for three different experiments performed at varying intensities. The parameters were determined using the Curve Fitting Toolbox application of MATLAB and are shown in Table 1. The obtained *R* ^2^ values were > 0.99 for all the model fits.

**TABLE 1:** Parameter values for the model fitted to the experimental formazan formation data.

Intensity [mW cm^−2^]	k’ [min^−1^]	k_3_ [nM. min^−1^]
56	0.004 ± 0.001	3 ± 2
101	0.012 ± 0.001	3.2 ± 0.3
455	0.026 ± 0.004	4.9 ± 0.4

Looking at the apparent first order rate constant k’, which can be considered a measure of the oxygen activation rate of the plasmonic rainbow photocatalyst as outlined above, a clear intensity dependence can be observed, as is expected for plasmon-driven photocatalytic reactions (Kale et al., 2014). The calculated rate constants for the zero order formazan discoloration reaction (*k*_*3*_) in Table 1 are of the same order of magnitude (nM.min^−1^) as those obtained in the earlier discoloration experiment (Figure 2D) and also display the trend of increasing k_3_ value at higher intensities. The combination of both calculated rate constants enables to interpret the peculiar behavior of the curves in Figures 2B, 3: The initial superoxide formation rate is higher at higher illumination intensities, but under these conditions the zero-order discoloration constant also increases. Due to the latter, the formazan concentration sooner reaches a maximum level after which the discoloration reaction dominates at high intensity values. Nonetheless, the excellent fits prove the validity of the kinetic model, which in turn enables to quantitatively evaluate the oxygen activation performance of different plasmonic systems by means of the apparent rate constant *k*’.

### 3.3 Plasmon-assisted oxidation of aniline to azobenzene

**FIGURE 4:**
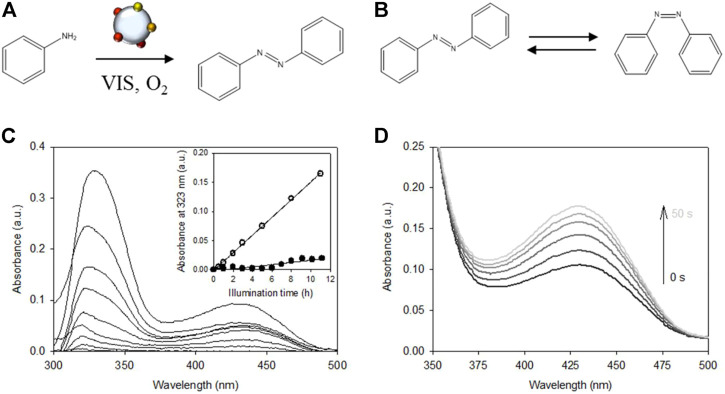
**(A)** Direct SPR mediated catalytic oxidation of aniline to azobenzene on SiO_2_ supported rainbow catalyst. **(B)** *trans* - *cis* isomerization of azobenzene. **(C)** Increase in UV-VIS absorption spectra of the illuminated reaction mixture hinting at azobenzene formation. Open circles in the inset show the corresponding linear increase of the absorbance at 323 nm as a function of illumination time (*R* ^2^ = 0.999). Filled circles represent the unilluminated dark experiment (*R* ^2^ = 0.908). **(D)** Change in the UV-VIS absorption spectrum of the final reaction product of **(C)** upon UV irradiation for 50 s. **(B)** shows the two isomeric conformations of azobenzene. The thermodynamically more stable *trans*-form (*t*-AB) can photo-isomerize to its corresponding *cis*-conformation (*c*-AB) under UV irradiation while the *cis*-to-*trans* back-isomerization occurs spontaneously in the dark. The absorption spectrum of *t*-AB in DMSO comprises a strong absorption at ∼323 nm (π→π^*^ transition) and a weaker band at ∼435 nm (*n*→π^*^ transition). The *cis* isomer has two bands in the UV, at ∼255 nm and ∼287 nm (both π→π^*^ transition), and one *n*→π^*^ transition at ∼435 nm that absorbs more strongly than *t*-AB (Bandara and Burdette, 2012; Hallett-Tapley et al., 2013).

To demonstrate the oxygen activation capacity of the plasmonic rainbow photocatalyst toward a relevant application, the plasmon-mediated oxidation of aniline to azobenzene under ambient conditions and visible light illumination is studied (Figure 4A), that exists both in a *trans*- and *cis*- configuration (Figure 4B). Aromatic azo compounds have found applications in different branches of industry as dyes, food additives, pigments, pharmaceuticals and precursors for natural product synthesis (Combes and Haveland-Smith, 1982; Kuwabara et al., 1999; Hunger, 2003; Cai et al., 2013; Ghosh et al., 2015). These compounds are conventionally made under high pressure and temperature. In addition, stoichiometric amounts of nitrite salts (entailing high amounts of inorganic salt waste) or environmentally unfriendly oxidants are required (Grirrane et al., 2008; Dutta et al., 2016; Peiris et al., 2016). Plasmonic metal nanoparticles provide an ecofriendly and sustainable alternative to these conventional synthesis methods as they allow the reactions to be carried out at less harsh reaction conditions using solely molecular oxygen as oxidant (Grirrane et al., 2008; Cai et al., 2013; Ghosh et al., 2015). A recent publication by da Silva et al*.* has shown that exploiting the plasmonic properties of gold and silver nanoparticles can lead to additional enhancements in the oxidation of aniline to azobenzene (da Silva et al., 2016).

For the aniline oxidation experiment, the catalyst powder was suspended in an oxygen saturated DMSO solution containing aniline and illuminated with the same visible light source as the one used in the XTT experiment (see Materials and Methods for more details). During the oxidation experiment, the samples showed a clear visual color change of the reaction mixture from colorless to faintly yellow, in accordance with the color of the pure *t*-AB (Figure 4C) (da Silva et al., 2016). The inset in Figure 4C shows that the changes of the most intense band (λ_max_ ∼323 nm) increase linearly with illumination time (open symbols). Similar to the XTT assay, blank measurements using unmodified SiO_2_ yielded no appreciable changes to the absorption spectrum. A different blank experiment performed in the presence of the rainbow catalyst but without illumination showed the appearance of the same bands but to a lesser extent (inset Figure 4C, filled symbols). These results agree with the ones obtained in the XTT assay leading to similar conclusions: (i) metal nanoparticles are responsible for the observed reactions and (ii) visible light induced plasmon excitation accelerates the reaction rate. From Figure 4D it can be seen that the absorption band at 435 nm increases under UV illumination. This fully supports *trans*→*cis* azobenzene isomerization (Figure 4B) as the absorption band at 435 nm of the *cis* isomer absorbs more strongly than *t*-AB. This increase in absorption was also reversible. These results indicate that a plasmon enhanced, noble metal catalyzed oxidative coupling reaction takes place during the performed aniline oxidation experiments. Additional experiments could be conducted to (i) check for possible by-product formation and (ii) quantitatively verify product conversion and selectivity. This, however, is outside the scope of this mere demonstration experiment.

### 3.4 Mechanistic considerations

**FIGURE 5:**
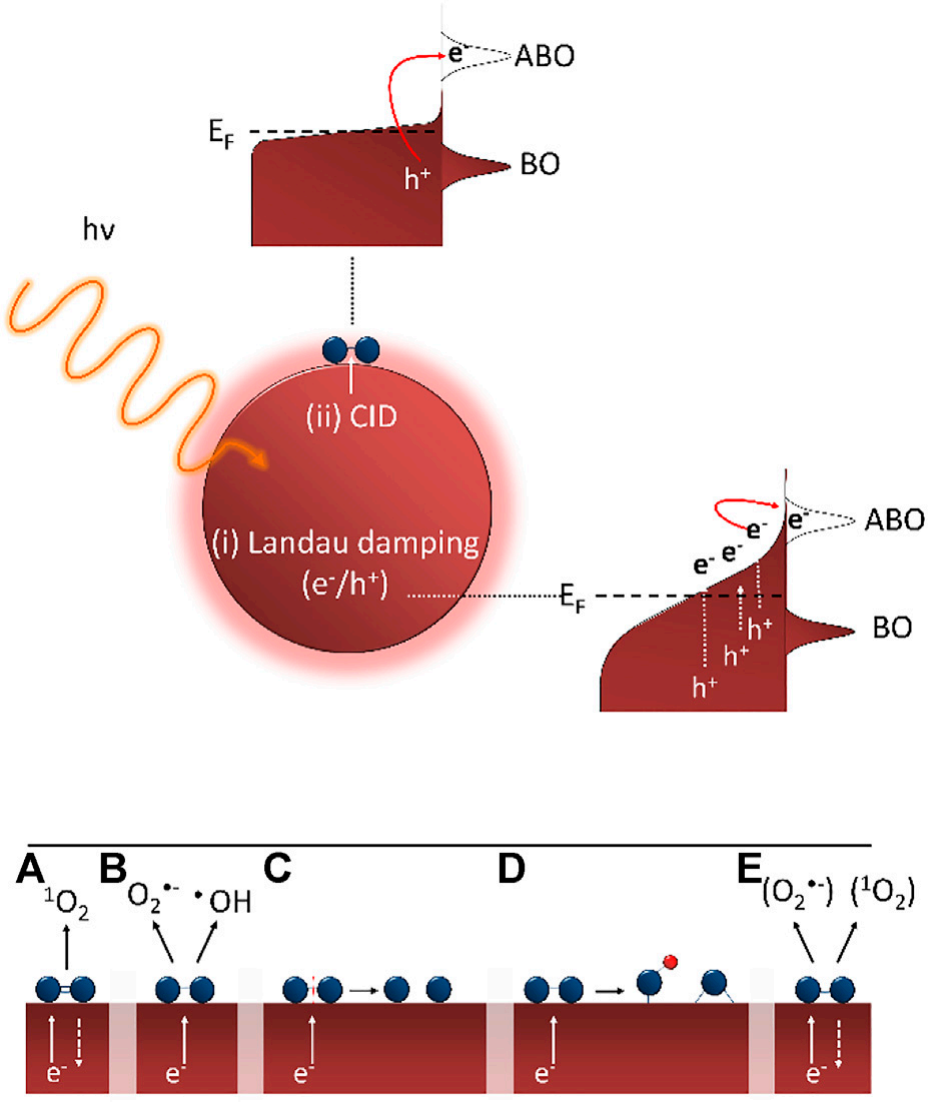
(top) Schematic representation of plasmon decay processes leading to indirect (i) or direct (ii) charge transfer of energetic electron to adsorbate (with ABO = antibonding orbital and BO = bonding orbital). Adapted from (Kale et al., 2014; Linic et al., 2015) (bottom) Proposed steps taking place after initial electron transfer: **(A)** singlet oxygen formation, **(B)** formation of superoxide and hydroxyl radicals, **(C)** desorption (reaction) induced by electronic transitions, **(D)** formation of surface oxides/hydroxides and **(E)** formation of superoxide-like and singlet oxygen-like species.

Different studies have shown that the first step in the direct oxygen activation on plasmonic nanoparticles is the formation of visible light induced energetic electrons that are subsequently transferred to adsorbed O_2_ molecules on the nanoparticle surface. Three main plasmon decay processes can be discerned: (i) radiative plasmon relaxation, (ii) non-radiative Landau damping resulting in electron-hole pair formation in the metallic nanoparticle and (iii) non-radiative chemical interface damping (CID) where an electron is directly injected in an adsorbate state. The radiative decay mechanism results in the reemission (scattering) of a photon and only becomes important for larger nanoparticles (Evanoff and Chumanov, 2004). The other two mechanisms are schematically represented in Figure 5 (top). The non-radiative Landau damping process results in the formation of energetic charge carriers in the nanoparticle that can in turn lead to chemical reactions on the nanoparticle surface *via* either localized heating or interaction of the formed charge carriers with adsorbed molecules (Linic et al., 2015). For the heating mechanism to have a significant impact on photochemical transformations, high light intensities (several orders of magnitude higher than the solar flux) and small nanoparticles (< 5 nm) are typically required, which is not the case in the present study. In the charge carrier-driven process, energetic charge carriers (electrons) directly interact with adsorbate states, forming transient negative ions (TNI) on the nanoparticle surface. The energetic charge carriers thus transiently interact with unoccupied adsorbate orbitals before the energy is dissipated to the surroundings. The adsorbate-nanoparticle complex is excited, inducing forces on the adsorbate thereby activating it and allowing chemical transformations to occur (Linic et al., 2015). Concerning this charge transfer of energetic electrons from the plasmon excited nanoparticle to the adsorbate, a distinction can be made between an indirect injection process [Landau damping, Figure 5 (i), top] and direct injection mechanism [CID, Figure 5 (ii), top].

The common feature of the abovementioned mechanisms is that plasmon excitation results in the formation of “hot” electrons that can subsequently occupy the antibonding π* orbital of adsorbed oxygen molecules, forming a TNI. The subsequent steps in this oxygen activation mechanism and the resulting (intermediate) chemical species are still unclear. Vankayala et al. (2011) were the first to observe that singlet oxygen (^1^O_2_) could be formed through plasmonic sensitization on gold, silver and platinum (Figure 5A). This plasmon-induced singlet oxygen formation was also observed by other research groups (Pasparakis, 2013; Gao et al., 2014). Singlet oxygen formation is proposed to be a two-step electron transfer process where first a “hot” SPR excited electron is transiently injected into the antibonding π* orbital of molecular oxygen, resulting in superoxide anion formation (O_2_−). This TNI subsequently relaxes by back transfer of the electron to the metallic nanoparticle, during which a spin-flip can occur yielding ^1^O_2_ (Vankayala et al., 2013; Gao et al., 2014). In addition to singlet oxygen, Gao et al. (2014) observed superoxide anion and hydroxyl radical formation on plasmon excited gold nanocages using dedicated EPR spin traps (Figure 5A,B). Linic et al. (2015) demonstrated that the reaction rate of ethylene epoxidation over 50 nm silver cubes is substantially increased under broadband illumination at elevated temperatures. They explained their results through the desorption (reaction) induced by electronic transitions (DIET) mechanism. Plasmon excitation results in the transfer of a “hot” electron into the antibonding orbital forming a TNI. This way energy is deposited in the O-O vibrational mode ultimately dissociating the adsorbed O_2_ molecule yielding atomic oxygen (Figure 5C) (Christopher et al., 2011).

The primary step in the reaction mechanism proposed by da Silva et al. (2016) for the SPR mediated oxidation of aniline to azobenzene on gold-silver nanoparticles is the generation of O_2_^−^ (da Silva et al., 2016). In our study, the presence of superoxide anions has indeed also been probed by the XTT assay.

## 4 Conclusion

The direct oxygen activation rate of a broadband plasmonic photocatalyst is evaluated by applying a probe assay based on the reduction of XTT to its corresponding colored formazan by superoxide. The use of an inert nano-SiO_2_ support enabled fine dispersion in liquid media, thereby combining advantages of homogeneous and heterogeneous catalysis. Visible light illumination substantially improved the oxygen activation efficiency due to the mediating role of plasmon excitation. A kinetic model is proposed to quantify the oxygen activation efficiency. An excellent match between model and experimental results is obtained. The plasmonic oxygen activation is also demonstrated towards an economic relevant application by monitoring the visible light-induced oxidation of aniline to azobenzene. UV-VIS analysis indicates that *trans*-azobenzene is formed *via* a plasmon-enhanced oxidative coupling reaction, that is commonly thought to proceed *via* intermediary O_2_^−^ species. We strongly hope the proposed superoxide probe-assay will become a facile and valuable tool to study also other related (plasmonic) oxygen activation reactions.

## Data Availability

The raw data supporting the conclusions of this article will be made available by the authors, without undue reservation.
