# Supplementary material for: Probing oxygen activation on plasmonic photocatalysts
Source: Front Chem. 2022 Sep 12;10:988542. doi: 10.3389/fchem.2022.988542 (PMC9510664; doi:10.3389/fchem.2022.988542)
Supplement: Supplementary file 1 [file DataSheet1.docx]

Supporting Information

Probing Oxygen Activation on Plasmonic Photocatalysts

Fons Dingenen^1,2^, Rituraj Borah^1,2^, Rajeshreddy Ninakanti^1-3^, Sammy W. Verbruggen^1,2*^

^1^Sustainable Energy, Air & Water Technology (DuEL), Department of Bioscience Engineering University of Antwerp, 2020 Antwerp, Belgium

^2^NANOlab Center of Excellence, University of Antwerp, 2020 Antwerp, Belgium

^3^Electron Microscopy for Material Science (EMAT), Department of Physics, University of Antwerpen, 2020 Antwerp, Belgium

*** Correspondence:**Sammy W. Verbruggen
[sammy.verbruggen@uantwerpen.be](mailto:sammy.verbruggen@uantwerpen.be)

1. Estimation of the XTT-formazan absorption coefficient at λ = 480 nm

Paull *et al*. (1988) have determined the molecular absorption coefficient (ε) of XTT-formazan to be 23823 M^-1^.cm^-1^ in water at 475 nm. In order to extract the equivalent value in DMSO as the solvent (dimethyl sulfoxide, Emplura, 99.0%), formazan was first synthesized using the ascorbic acid reduction method in water. Here, 27.1 mg of ascorbic acid (Sigma-Aldrich, [≥99.7%](https://www.sigmaaldrich.com/BE/en/product/sigald/s6014)) was mixed with 25.9 mg of sodium bicarbonate (Sigma-Aldrich, [≥99.7%](https://www.sigmaaldrich.com/BE/en/product/sigald/s6014)) in 15 mL of demineralized water. 10 mg of XTT (2,3-bis(2-methoxy-4-nitro-5-sulphophenyl)-5-[(phenylamino)carbonyl]-2H-tetrazolium, sodium salt, Chem-Lab, ≥ 85%) was added to 1.5 mL of this solution. The resulting suspension was stirred at room temperature for several days in the dark forming a dark red solution. Afterwards, the reaction mixture was diluted with 4.5 mL of ethanol to form a thick red suspension (Paull et al., 1988). Finally, 10 µL of this suspension was dissolved in a cuvette containing 0.99 mL of either water or DMSO. Since 99 vol% of the cuvette medium is water or DMSO, the measured spectrum can be considered as the formazan absorbance in that respective solvent. A dilution series with formazan concentrations up to 0.25‰ was established for both the water and the DMSO solvent (Figure S1A). Note that a slight red-shift can be observed when comparing formazan and water, with maxima around 480 and 475 nm, respectively (Figure S1B). Consequently, the molecular absorption coefficient reported in water is corrected for the one in DMSO according to:

$\varepsilon_{DMSO, 480 nm}= \frac{slope in DMSO}{slope in water}. \varepsilon_{water, 475 nm}$ (1)

This results in a corrected absorption coefficient, ε_DMSO, 480 nm_ of (26.1 ± 0.3).10^3^ M^-1^.cm^-1^.


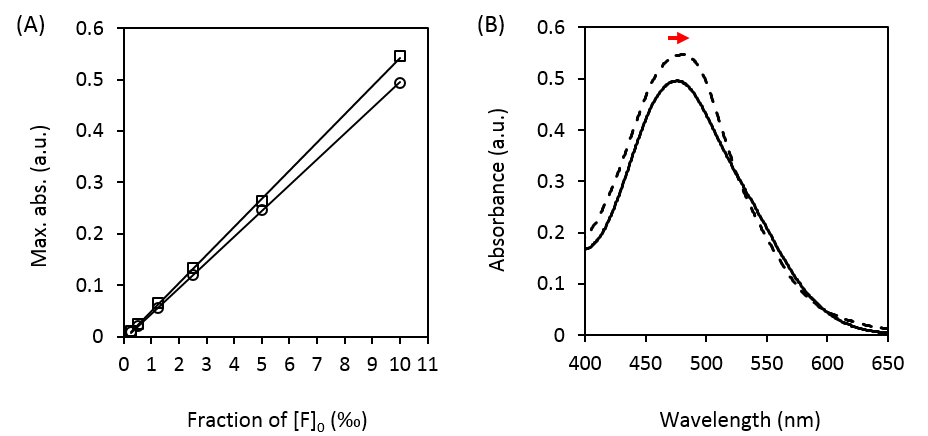


**Figure S1.** (A) Formazan dilution series in DMSO (□, slope of 0.0545 ± 0.0004) and water (○, slope of 0.0498 ± 0.0003). (B) UV-VIS absorption spectrum for 1% formazan solutions in water (full) and DMSO (dashed line). Note that the small red-shift is indicated with a red arrow.

2. Kinetic model

The model is based on the one proposed by Oritani et al*.*, who studied the electron transfer between water soluble tetrazolium salts and superoxide (Oritani et al., 2004). The goal is to obtain a model that can be used to fit the observed trends in formazan evolution during the XTT assay. From these fits relevant and quantitative information on the oxygen activation efficiency of the catalyst under study can then be extracted. The general idea behind the XTT assay is that plasmon excited nanoparticles activate oxygen by transferring a ‘hot’ electron to adsorbed O_2_ molecules on the nanoparticle surface thereby forming O_2_^•-^. This superoxide anion radical subsequently reacts with XTT forming formazan (F), of which the formation can be monitored using UV-VIS spectroscopy. The reactions are summarized in Equation 2 and 3.

$Rainbow photocatalyst on SiO_{2}+O_{2}\underset{\to}{h\nu}O_{2}^{\bullet-}$ (2)

${2O}_{2}^{\bullet-}+XTT\underset{\to}{H^{+}}2O_{2}+F$ (3)

The reduction of tetrazolium salts occurs via a two-electron process with the intermediary formation of a one-electron reduced tetrazolinyl radical. This one-electron reduced tetrazolium salt radical subsequently disproportionates into the original tetrazolium salt and its corresponding formazan (*i.e.* the two-electron reduced form).(Neugebauer and Russel, 1968; Bielski et al., 1980; Viseu et al., 1990; Oritani et al., 2004) A possible scheme for this reaction step is shown in Figure S1.

Based on the reaction mechanism shown in Figure S2, Equation 2 can be further subdivided into two elementary reactions (Equation 4 and 5). In addition, the zero-order photo-induced discoloration of the formed formazan product invokes the need for Equation 6, with F_d_ being a colorless formazan derivative.

$O_{2}^{\bullet-}+XTT\underset{\to}{k_{1}}O_{2}+{XTT}^{\bullet}$ (4)

${2XTT}^{\bullet}\underset{\to}{k_{2}, H^{+}} XTT+F$ (5)

$F\underset{\to}{k_{3}, h\nu} F_{d}$ (6)

Assuming that the rate of change of the intermediate tetrazolinyl radical is zero (steady-state approximation), the kinetic equations can be simplified ultimately yielding the formazan formation rate described by Equation 7.

$\frac{d\left[ F \right]}{dt}=k_{1}\left[ O_{2}^{\bullet-} \right]\left[ XTT \right]-k_{3}$ (7)

Prior to each photoinduced oxidation reaction, the airtight sealed reaction vessel containing the plasmonic catalyst and probe molecule in DMSO, was saturated with oxygen. Since the catalyst is then illuminated during the entire experiment it is fair to assume that [O_2_^•-^] >> [XTT]. The product of k_1_ and [O_2_^•-^] can therefore be considered constant during the reaction, and is replaced by an apparent first order rate constant k’ (Equation 8). k’ thus only represents a quantitative measure for the oxygen activation efficiency of the plasmonic photocatalyst since k_1_ is a catalyst-independent constant for the general reaction between the superoxide anion and XTT. Thus, the greater k’, the more superoxide is formed, and the higher the catalytic activity. During the experiment, the sum of XTT and all its related species (XTT^•^, F and F_d_) can also be considered constant and equal to the initial XTT concentration [XTT]_0_ (principle of conservation of mass). In addition, given the zero-order formazan discoloration, [F_d_] can be rewritten as k_3_t ultimately yielding Equation 9.

$k^{'}=k_{1}\left[ O_{2}^{\bullet-} \right]$ (8)

$\left[ XTT \right]_{0}=\left[ XTT \right]+\left[ F \right]+k_{3}t$ (9)

Substituting Equations 7 and 8 in Equation 6, the following integrated rate equation is obtained (Equation 10):

$\left[ F \right]=\left( \left[ XTT \right]_{0}-k_{3}t \right)\left( 1-e^{{-k}^{'}t} \right)$ (10)

Equation 10 thus represents a kinetic model for the formazan concentration, [F], as a function of time, that can be monitored using the absorption band at 480 nm and the molecular absorption coefficient, estimated in 1. In addition, the initial XTT concentration, [XTT]_0_, can also be described with this absorption band. Assuming no discoloration of the formed formazan would take place, the formazan concentration at t = infinity equals [XTT]_0_ as all XTT would be converted into formazan. [XTT]_0_ can therefore be substituted by the absorbance of formazan at 480 nm at t = infinity (in the absence of discoloration), ultimately yielding (eq. 11).

$\left[ F \right]= \frac{{Abs}_{480 nm}\left( t=t \right)}{\varepsilon_{DMSO, 480 nm} . l}=\left( \frac{{Abs}_{480 nm}\left( t= \infty\right)}{\varepsilon_{DMSO, 480 nm}. l}-k_{3}t \right)\left( 1-e^{{-k}^{'}t} \right)$ (11)

With ε_DMSO, 480 nm_ the estimated molecular absorption coefficient for formazan and l the path length of light through the solution (1 cm).

**
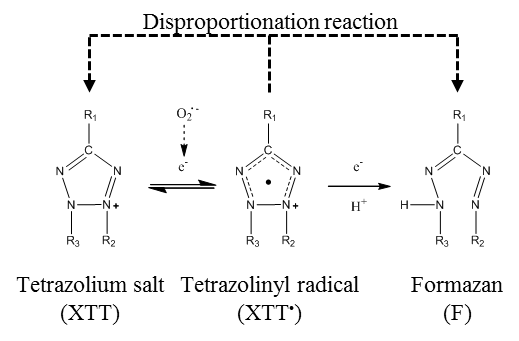
**

**Figure S2.** Reaction mechanism of the reduction of a tetrazolium salt (XTT) to the one-electron reduced intermediate radical (XTT•) and its two-electron reduced formazan form. Adapted from ref (Oritani et al., 2004) .

**References**

Bielski, B. H. J., Shiue, G. G., and Bajuk, S. (1980). Reduction of nitro blue tetrazolium by CO2- and O2- radicals. *J. Phys. Chem.* 84, 830–833. doi: 10.1021/j100445a006.

Neugebauer, F. A., and Russel, G. A. (1968). Tetrazolinyl Radicals. *J. Org. Chem.* 33, 2744–2746.

Oritani, T., Fukuhara, N., Okajima, T., Kitamura, F., and Ohsaka, T. (2004). Electrochemical and spectroscopic studies on electron-transfer reaction between novel water-soluble tetrazolium salts and a superoxide ion. *Inorganica Chim. Acta* 357, 436–442. doi: 10.1016/j.ica.2003.05.007.

Paull, K. D., Shoemaker, R. H., Boyd, M. R., Parsons, J. L., Risbood, P. A., Barbera, W. A., et al. (1988). The synthesis of XTT: A new tetrazolium reagent that is bioreducible to a water-soluble formazan. *J. Heterocycl. Chem.* 25, 911–914. doi: https://doi.org/10.1002/jhet.5570250340.

Viseu, M. I., Gonçalves, M. L. S. S., Costa, S. M. B., and Ferreira, M. I. C. (1990). Mechanism of the electrochemical reduction of tetrazolium blue in non-ionic micelles. *J. Electroanal. Chem.* 282, 201–214. doi: 10.1016/0022-0728(91)85099-B.
